# Attitudes towards and health consequences of female genital mutilation/cutting: A qualitative study among Somali and Kurdish immigrants and descendants in Denmark

**DOI:** 10.1016/j.eurox.2024.100315

**Published:** 2024-05-17

**Authors:** Ditte S. Linde, Hawa-Idil Harakow, Negin Jaafar

**Affiliations:** a Department of Clinical Research, University of Southern Denmark, Odense, Denmark; b Department of Gynaecology and Obstetrics, Odense University Hospital, Odense, Denmark; c Department of Surgery, Esbjerg and Grindsted Hospital, Esbjerg, Denmark; d Department of Gynaecology and Obstetrics, Aarhus University Hospital, Aarhus, Denmark

**Keywords:** Female genital mutilation, Sexual and reproductive health, Immigrants, Stigma, Sexual trauma, Cultural sensitivity, SDG5

## Abstract

**Objectives:**

There is lack of in-depth knowledge of how immigrants who originate from countries where female genital mutilation/cutting (FGM/C) historically is practiced, perceive the practice after migrating to Europe. The aim of this study was to explore the attitude towards FGM/C among immigrants and descendants and the health consequences of living with FGM/C.

**Study design:**

Qualitative methods were used in the form of semi-structured interviews and focus group discussions. Female and male immigrants and descendants in Denmark from Somalia or Kurdish of Iraq/Iran participated in the study. The interview/focus group discussion guides were developed by the European Institute for Gender Equality. Purposely sampling was used, and participants were recruited by use of snowballing through gatekeepers and women’s societies working within the Somali and Kurdish communities.

**Results:**

Sixteen persons participated in the study. No descendants had been cut, but all female immigrants had been cut prior to migrating and did not wish to pass on the practice. FGM/C was perceived as a harmful practice with severe sexual and mental health consequences. Women with Somali origin experienced that the practice was falsely associated with their origin, which led to stigmatisation. Women with Kurdish origin lacked healthcare support when suffering sexual consequences of FGM/C. It was generally perceived that the Danish healthcare system lacked cultural sensitivity.

**Conclusion:**

FGM/C is negatively perceived among Somali and Kurdish immigrants and descendants in Denmark and not practiced among these groups. The Danish healthcare system should adopt a more culturally sensitive approach when addressing sexual health among immigrants and descendants. Denmark and other European countries should work towards destigmatising the immigrant communities when it comes to FGM/C. Larger European studies with primary data are needed to generalise the findings of this study.

## Highlights

• FGM/C is negatively perceived among Somali and Kurdish immigrants and descendants in Denmark.
• This first Danish study with primary data, unlike European studies using extrapolated data, indicates that FGM/C is not practied in Denmark.
• Somali immigrants and descendants in Denmark feel stigmatised as FGM/C is falsely associated with their origin.
• European countries should destigmatise immigrant communities in regards to FGM/C and conduct more studies with primary data.
• Kurdish women cut before migration lack support for sexual consequences of FGM/C. The Danish healthcare system should adopt a culturally sensitive approarch.

## Abbreviations

EIGE: European Institute for Gender Equality
EU: European Union
FGM/C: Female genital mutilation/cutting
WHO: World Health Organization

## 1 Introduction

Female genital mutilation/cutting (FGM/C) is a term that refers to all procedures that involve partial or total removal of external female genitalia or other injuries to the female genitalia for non-therapeutical reasons [1]. The World Health Organization (WHO) distinguishes between four types of FGM: (I) Clitoridectomy – partial/total removal of the clitoris; (II) Excision – partial/total removal of clitoris and Labia minora with/without excision of labia majora; (III) Infibulation – narrowing of the vaginal orifice by creating a covering seal by cutting/appositioning Labia minora and/or labia majora with/without excision of clitoris; and (IV) all other harmful procedures to the female genitalia for non-medical purposes [2]. FGM is a custom that has been widely practiced for centuries in over 30 countries worldwide extending from regions in Africa to the Middle-East and Asia [3]. The WHO estimates that more than 200 million women and girls have undergone FGM, and more than three million girls are in risk of FGM annually though the actual number is unknown due to lack of reliable data [4]. FGM is internationally recognised as a human rights violation, and in 2012 the United Nations General Assembly adopted a resolution to end FGM [5]. Further, the global commitment to end FGM was reaffirmed by the Sustainable Development Goals where target 5.3 calls to eliminate FGM by year 2030 [6].

FGM is associated with immediate and long-term consequences to women’s physical, mental, sexual, and reproductive health. Immediate physical health consequences of FGM include severe pain, infection and haemorrhage, while the long-term physical health consequences include scarring, keloid formation, chronic pain, genitourinary complications [7]. Further, obstetric complications include episiotomy, prolonged labour, and emergency caesarean section [8]. The sexual consequences of FGM may entail dyspareunia, vulvodynia, and sexual arousal disorder, orgasmic disorder, satisfaction, and feeling inadequate during intimacy with their partner [9], [10], whilst mental health consequences include depression, anxiety, post-traumatic stress disorder, low self-esteem, and feeling shameful about the appearances of one’s genitalia [10], [11].

Globalisation, transnational migration and forced displacement of persons have caused the issue of FGM to cross borders and thus becoming a global health issue that governments and healthcare professionals must deal with in all parts of the world, including Europe. In Denmark, a subgroup of the migrant population originates from countries where FGM is practiced, hence, several female immigrants (i.e., first-generation immigrants) live with the consequences of FGM, as they have a been subjected to FGM prior to migrating to Denmark. Further, several female descendants (i.e., second-generation immigrants) may be in risk of FGM, if the practice is still a cultural norm among their parents or extended family. In Denmark, the largest population groups who originate from FGM-practicing countries are Somalians and Kurdish Iraqis with the vast majority being descendants [12].

Several European studies have tried to estimate the prevalence of FGM in an European context [13], [14], [15], and since 2012, the European Institute for Gender Equality (EIGE) has mapped the situation of FGM in the European Union (EU) by compiling national data from different EU countries to estimate the number of girls in risk of FGM. To date, four EIGE reports have been made involving 13 EU member states, and the latest study from 2021 included Denmark [12]. However, due to the lack of national systematic representative surveys, the EIGE reports and other European studies mainly estimate the prevalence and number of migrant girls in risk of FGM indirectly by extrapolating demographic health survey data of migrant populations from FGM-practicing countries. Yet, this method may not accurately emulate the magnitude of the issue as migrant populations may not be representative of the population in their country of origin. Further, the immigration process may have influenced their cultural norms and attitudes towards the practice [16], [17]. Qualitative studies among immigrants and descendants that provide in-depth data of the attitudes, health consequences, and healthcare support of FGM in Europe are therefore of key importance. Such studies will provide first-hand accounts of the issue of FGM and may more accurately describe the magnitude of the issue as well as nuance the understanding of FGM in a European context. EIGE’s latest reports also include a qualitative component where perceptions towards FGM among migrant populations were explored, though this was not the main focus of the report [18].

### 1.1 Aim

This paper aims to explore the attitude towards the practice of FGM in a Danish context among immigrants and decedents in Denmark who originate from countries where FGM is practiced. Further, the study aims to explore the health consequences of FGM among female immigrants living with FGM in Denmark.

### 1.2 Study context

The data were collected as part of EIGE’s report on the estimation of risk of female genital mutilation in four EU countries, i.e., Denmark, Spain, Luxembourg, and Austria [12]. Key elements of the qualitative study were represented in the report, yet the report did not make an in-depth analysis of the qualitative results and did not describe findings that went beyond the overall focus of the report. Hence, these are to be reported in this article in agreement with EIGE.

## 2 Material and methods

### 2.1 Study design and participants

This was a qualitative study, and the target group was major immigrant groups and descendants in Denmark who stem from countries where FGM is practiced. The data were collected by use of personal semi-structured interviews and focus group discussions with (1) male and (2) female immigrants originating from Somalia, (3) female descendants from Somalia, and (4) female immigrants from Kurdistan (Iraq and Iran) residing in Denmark. Originally, the study was planned to solemnly consist of focus groups discussions, however, due to the sensitive nature of the topic, some participants preferred to participate through personal interviews. Female immigrants and descendants from Somalia participated in focus group discussions, yet one female descendant did not show up for the focus group discussion and were interviewed personally afterwards. The remaining participants were personally interviewed. Further, all data were scheduled to be collected face-to-face but due to the COVID-19 pandemic parts of the data collection were conducted online, i.e., the focus discussion with female Somali descendants and the personal interviews.

The interview guides were semi-structured and developed by EIGE in English. There was a separate interview guide for each target group, and prior to the study starting the interview guides were translated from English into Danish by the primary investigator. Non-governmental organisations and experts within the field of FGM commented upon the interview guide prior to the study starting and based on their input the interview guide was revised. Further, the primary investigator (first author) was experienced in conducting qualitative research and participated in a qualitative training session with European fellows commissioned by EIGE prior to data collection starting.

### 2.2 Data collection and analysis

Data were collected between 1 October and 14 November 2020. Purposively sampling was used to reach female immigrants who had been subjected to FGM/C prior to migrating as well as male immigrants and descendants who originated or descended from countries where FGM/C is historically practiced. Participants were recruited by use of snowballing where gatekeepers and women’s organisations working within the immigrant environment in Denmark were contacted, who then reached out to the target group and asked if they could be interested in participating in the study. The focus group discussion and interview with descendants from Somalia were conducted in Danish by the whilst the focus group discussion with female immigrants from Somalia were conducted in Danish with direct translation from Somali to Danish where needed. The personal interviews with male Somali immigrants were conducted in Danish whilst the interviews with Kurdish immigrants were conducted in Kurdish with direct translation to Danish. Further, all focus group discussions and interviews were audio-taped and thorough notes were taken. The first author conducted all focus group discussions/interviews whilst the second author served as translator and notetaker in the Somali focus group discussions, and the third author served as gatekeeper and translator in the interviews with Kurdish women. No one else were present during the interviews/focus group discussions. All data were transcribed and summarised, imported into NVivo 12, and a content analysis was conducted in two steps. Firstly, deducted coding was conducted by the first author by use of a coding frame with overall themes developed by EIGE. Secondly, all transcripts were reread by the first and second author, and data were recoded using an inductive approach where all themes and codes arose from the transcripts. Finally, the deductive analysis was compared with the inductive analysis, and supplementary themes were added to the original analysis plan. To avoid the translation link, ‘she’ was replaced with ‘I’ in the transcripts.

### 2.3 Patient and public involvement

After the study had finished and data had been analysed, all participants were invited to comment on the overall study findings.

### 2.4 Ethics

Prior to the study starting, the Regional Committees on Health Research Ethics for Southern Denmark were contacted in order to clarify whether or not ethical clearance was needed for the study to be conducted. The Committees decided that no ethical clearance was needed to conduct the study, which is in line with the Danish Committee Act (§14, unit 2) [19]. However, a due to the sensitive nature of the topic, a number of ethical considerations were considered throughout the study. All participants gave written and verbal consent prior to participating in the study, and all participants were informed that they could withdraw from the study at any time, and if they felt that any questions were uncomfortable, they did not have to answer. In case participants felt distress during the interviews/focus group discussion, this was acknowledged, and the person was given time and asked if they wanted to proceed or stop. This occurred during some of the interviews with Kurdish immigrants. Data (audio recordings and transcripts) were stored safely on a secure drive at the University of Southern Denmark. As a gift of gratitude, all participants were offered a voucher of DKK 200 (∼ Euro 27) to a voluntary Danish store. To ensure confidentially of all participants and to hide their identity, all participants were anonymised. The study is reported according to the Standards for Reporting Qualitative Research checklist (Supplementary file I) and in accordance with the Helsinki declaration.

## 3 Results

### 3.1 Participant characteristics

Overall, the recruitment for the study was difficult due to the sensitive nature of the topic and because there is no organised network working with FGM in Denmark. Further, there was a general fear – especially among the Somali immigrants and descendants – of being associated with FGM/C, which led them to decline to participate in the study. A total of 16 persons participated in the study; five female Somali immigrants, four female Somali descendants, three male Somali immigrants and four Kurdish immigrants, of which three came from Iraq and one came from Iran. The interviews lasted between 25 min and 2 h, whilst the focus group discussions lasted between 1,5 h and 2 h. The age of the Somali descendants ranged between 22 and 32 years whilst the age range of the immigrants ranged between 27 and 56 years. All participants were Muslim except one Kurdish woman who was converted Christian. The female immigrants had all been mutilated prior to immigrating to Denmark whilst none of the descendants had been cut (Table 1).

**Table 1:** Characteristics of study participants.

Socio-economic characteristic	Kurdish female immigrants	Somali female immigrants	Somali male immigrants	Somali female descendants
Age (mean [min-max])	46 [27–56]	40 [30–44]	43 [38–52]	26 [22–32]
**Marital status**				
Married	3	1	3	2
Engaged	1	-	-	-
Single	-	-	-	2
Divorced	-	4	-	-
**Educational status**				
Primary school	2	3	-	-
Secondary school	2	2	2	-
University	-	-	1	4
**Number of children**				
0	0	-	-	3
1	-	-	-	1
2	1	1	-	-
3	2	1	1	-
4 +	-	3	2	-
**Religion**				
Muslim	3	5	3	4
Christian	1	-	-	-
**Number of years living in Denmark**				
Grew up in Denmark	-	-	-	4
0–5	1	-	-	-
6–15	-	-	-	-
16–20	2	-	-	-
20 +	1	5	3	-
Total	4 (100 %)	5 (100 %)	3 (100 %)	4 (100 %)

### 3.2 Attitudes towards FGM/C

Overall, there was wide agreement across all groups that FGM/C was an outdated practice that should be eradicated globally. For example, participant #1 said, “*The culture has changed – people know it is wrong”* [participant #1, Kurdish immigrant] whilst another stated that, “*I would never do it to my daughter […] and even if I wanted to, my daughter would not accept it. But I think like my daughter – it [ed. FGM] should not be repeated”* [participant #2, Kurdish immigrant]*.* A Somali male immigrant, put it as such,

*“People care about their children – violence and cutting are old fashioned. It has a lot of consequences. […] I often drink tea with men at praying halls, and I have never heard anyone speak about keeping the practice. It is disappearing […] it is becoming extinct.”*

[participant #3, Somali immigrant]

### 3.3 Consequences of FGM/C

Both women and men were highly aware of the sexual and psychosocial consequences of FGM/C of women. However, there was a difference in attitude to the extent that the experience still affected them, among the women who had been subjected to FGM/C prior to immigrating to Denmark. The Somali immigrants felt that this was a negative aspect in their life that they just had to “deal with” and could do nothing about, and their main priority was to ensure that this practice was not passed on to the next generation so that they would not have to suffer the same consequences as them. In contrast, all the Kurdish women felt that this was a trauma that still affected their lives to a high degree. Especially the sexual consequences were a major issue in their relationships. For example, a woman described that she felt *“half and not whole”* [participant #4, Kurdish immigrant] whilst another said that,

*“I have never* experienced *sexual pleasure […] I am like a solider when I am with a man – I do not feel anything. I am a living dead […]”*

[participant #2, Kurdish immigrant]

Yet another woman described how the experience of being cut had left a constant fear of seeing healthcare professionals, which caused her not to seek help,

“*The experience was so traumatic for me, that I cannot overcome to see a doctor and see a knife again even though I want to get help”*

[participant #5, Kurdish immigrant]

### 3.4 Practice of FGM and virginity

All participants agreed that FGM is not practiced in Denmark, and that the younger generations (i.e., second and third generation girls) are not in risk of undergoing FGM, neither in Denmark nor in the countries which their parents originated from. For example, a woman said that *“I have lived in Denmark for 28 years, and I have never heard about anyone […] cutting their daughters”* [participant #5, Kurdish immigrant].

Chastity was still considered important by most Somali and Kurdish participants, however, they agreed that this should be achieved through education and not control. For example, a participant said that,

*“It [ed. virginity] is still a central part of one’s upbring, but FGM is now out of the equation. It is more dialogue-based where you advice young girls against doing it [ed. having sex prior to marriage]. Both in Denmark and in Somalia. Today you cannot ensure it [ed. virginity]. […] Of course, there are some who ‘fall in’ [ed. have sex prior to marriage], but it is up to the individual. I find value in waiting until one is married. My whole family does”*

[participant #6, Somali Descendant]

Whilst another woman described it as such, *”Sex prior to marriage is ‘haram’. It is forbidden – it is a no go. […] [ed. And] I am against cutting [ed. FGM]. […] Whether you take the Christian path or the Muslim [ed. FGM is forbidden]. What separates us is sex prior to marriage”* [participant #2, Kurdish immigrant].

All participants also agreed that FGM largely had been a “women’s practice” that back in the days had been carried out by elderly women towards younger girls. Hence, the change in attitude towards ending the practice had been part of a larger woman’s movement, and women had played a crucial role in changing the mindset towards FGM. A participant described it this way*,* “*My mother was very vocal about her choice of not having me and my sister cut, and my family respected that”* [participant #6, Somali descendant] whilst another said that “*It is my mother who decided [ed. about the practice] […] and she did not want it. It is important with a strong mother – our mothers are our executive directors. […]”* [participant #7, Somali Descendant].

The Kurdish women expressed that the practice was already on its way to being abandoned in the 1990s in Kurdistan, therefore they did not currently see it as an issue. Further, the Somali participants stated that Somalis had abandoned the practice upon arrival to Denmark, and several of the descendants also described how their mothers had been involved in the now former Danish non-governmental organisation “Society against FGM” which had advocated strongly towards ending the practice. Thus, they had played a significant role in ending the practice among Danish-Somalis. It was also pinpointed by men that there had been a change of attitude within Somalia, which had led to a shift in practice in Somalia,

*”It used to be practiced, but there has been a major effort against it in the big cities in Somalia. […] [ed. Through] workshops and* via *development aid, but also religious courses and ceremonies. It is forbidden according to Islam. […] It is not practiced in Denmark and in their [ed. immigrants] home country. Women are no longer [ed. being cut]. Then they have to go rural Somalia. […] It [ed. FGM] has first place among the things that you want to prevent.”*

[participant #8, Somali immigrant]

### 3.5 Stigma

The Somali participants expressed a great deal of frustration with the practice continuously being associated to their Somali roots as they had abandoned the practice long ago – they felt it was a false narrative. The participants expressed that Danish-Somalis generally were portrayed very negatively in the media, and that the discourse about FGM/C was a part of that portrayal. This led to a reluctancy to discuss topic publicly which led to it becoming a taboo in society. A participant described it this way,

*“Girls do not want talk about it* due *to stigma. They are afraid that it will lead to a negative debate in the media about Somalis and FGM”*

[participant #9, Somali immigrant]

Further, the younger women experienced stigma during consultations with healthcare professionals. For example, a Somali descendant stated that a doctor had asked her, *“You are not one of those girls who have been cut, are you?” [participant #10, Somali descendant].* The issue of stigma towards Somalis were also present in the interviews with the Kurdish women, who also had the impression that FGM/C was still practiced among some Somalis. For example, a participant said that *“[…] I have heard about Somali girls living in Denmark, who are being taken back to Somalia to be vandalised. I have read about it on the internet and heard it in the media”*[participant #5, Kurdish immigrant]. According to the Somali participants, the association between Somalis in Denmark and FGM/C is a fallacy as the practice has no relevance nor implications for young girls today. Hence, there was a call for a cessation of the supposed correlation between FGM and Somali immigrants in Europe.

### 3.6 Lack of healthcare support for women living with FGM in Denmark

FGM/C was described as a very taboo topic – both inside the communities as well as in the general society. Whilst the Somali female immigrants – who had been cut prior to coming to Denmark – felt no need for healthcare support to deal with the consequences of FGM/C, the case was different for the Kurdish women. In contrast, they would like help to deal with the long-term sexual and psychological consequences of FGM/C but felt that it was impossible for them to get any health care support. A participant described the issue as such,

*“I have not told any doctors [ed. about being cut], and they have never asked […] [ed. but] of course I* would *be honest if they asked”*

[participant #4, Kurdish immigrant]

Another woman described her need for help as such,

*“I just want help – pills, a creme, medicine which can help me. I am too young to live like this for ever”*

[participant #1, Kurdish immigrant]

For some women, the language barrier was described as a big obstacle for seeking help and several women stated that it was impossible for them to discuss the issue when they did not speak the same language as the healthcare personnel. For example, a participant said that *“I am grateful for the healthcare system, but the biggest hurdle is my language issue. […] I could have received more help after giving birth [ed. but] I was not seen or heard” [participant #2, Kurdish immigrant].*

### 3.7 Lack of cultural sensitivity within the Danish healthcare system

All groups agreed that there was a need for increased cultural sensitivity within the Danish healthcare system, even beyond a topic as sensitive as FGM. A participant expressed it the following way,

*“There are lot of different ethnicities in Denmark, and we should be better at educating the health personnel to understand the challenges they have”*

[participant #9, Somali descendant]

Some women expressed a need for healthcare professionals with same cultural/ethnic background as themselves, as they felt very isolated in the Danish society and thought it would make consultations easier and more comfortable. Further, several participants also advocated for a bigger focus on how to communicate well with immigrants within the Danish healthcare system – especially when it came to discussing sensitive issues such as sexual health. For example, a participant stated that,

*“This is not a new issue for the Danish healthcare system. We should improve how we communicate about it [ed. because] you do not know if you are in ‘safe hands’[ed. when discussing sexual health issues with a Danish doctor]”*

[participant #7, Somali Descendant]

## 4 Discussion

This study showed that there is a negative attitude towards FGM/C among Somali immigrants and descendants and Kurdish immigrants in Denmark, and that FGM/C is not practiced in a Danish context. Further, the study showed that the Somali groups are stigmatised when it comes to FGM/C as they are still associated with the practice even though they have abandoned the practice. Women with Kurdish origin, who live with FGM/C, would like help from the Danish healthcare system to deal with sexual consequences of FGM/C.

### 4.1 Strengths and limitations

A major strength of this study is that it is the first study from Denmark with first-hand accounts of the attitude towards FGM/C – and the consequences of living with FGM – among Kurdish and Somali immigrants and descendants. Due to the sensitive nature of FGM, there are generally very limited European studies with first-hand accounts from immigrants and descendants, hence, this study contributes with important and nuanced insights into how European immigrants and descendants – who stem from countries that practice FGM – perceive the practice and live with the consequences. Hence, our findings may nuance the public debate in Denmark specifically – and Europe overall – about the extent of the issue as well as how to address sexual health issues among immigrants who live with FGM.

A limitation of all qualitative studies is they have small samples sizes as the aim of them is to get in-depth perspectives of the life-worlds of selected groups rather than being powered to detect significant statical differences and conduct subgroup investigations within larger populations. Therefore, data collection stops when data are saturated, rather than when a certain (larger) number of participants have been included into the study as in quantitative studies. Hence, a major limitation of our study is the small sample size and we cannot rule out that our study population may not be representative to the broader population of Somali and Kurdish immigrants in Denmark, i.e., our results may not be generalisable. Quantitative or mixed-method studies with a larger and more diverse sample are therefore needed as these would enhance the generalisability of the findings. Further, another limitation of this study is that Kurdish men were not included in the study, hence, future studies that also investigate the perspectives of Kurdish men should be conducted in order to see if their perception of FMG/C is in line with immigrant women with a Kurdish background and immigrant men with a Somali background. Additionally, we cannot rule out selection bias in our study. Recruitment was extremely difficult, the majority of participants came from Eastern Jutland, and many participants knew each other from their local community, hence, it is possible that the beliefs about FGM/C would differ if participants had been recruited from other immigrant communities in Denmark and that this study only captures certain elements of the perception of FGM/C among immigrants and descendants in Denmark. That being said, we expect this issue to be minor as other participants from other parts of Denmark declined to participate in the study as they feared being associated with FGM/C because they were very much against it.

### 4.2 Comparison with existing literature

There is limited literature to compare our study findings to, however, those few studies that are comparable, overall support our findings. A 2023 study from the United Kingdom (UK), which reviewed how data are compiled to estimate girls in risk of FGM/C in the UK, found that the estimated number of girls in risk are over-inflated as they are based on extrapolated that from a selection of high FGM/C-prevalence countries, and the reliability of these data – even in those countries where the data are collected – are questionable. This over-estimation harms communities and contributes to institutional discrimination and racially/religiously-motivated victimisation of innocent families [20]. The findings of this study was supported by a 2023 comment from Sweden, which argued that these findings go beyond the UK, and that the overblown risk and prevalence estimates that European policymakers and the mass media use are seriously flawed, which leads to discrimination [21]. This supports our finding of stigmatisation and discrimination of Somali immigrant groups in Denmark and suggests that there is a need for European countries to work towards destigmatising immigrant communities in relation to FGM/C. One approach could be to conduct more studies that make use of primary data – such as qualitative studies such as ours. Further, stigmatisation could be reduced if studies more accurately estimate the prevalence and risk of FGM/C. e.g. through national surveys, as well as expand the understanding of FGM/C and sexuality in a wider degree among public institutions and healthcare professionals in partnership with the immigrant communities. Our finding that FGM/C is negatively perceived among immigrants and descendants and not a practice that should be passed on the next generation is also supported by other qualitative studies from Europe. A 2022 study from the Netherlands found that migrant populations with origin from various FGM/C-practicing countries had no intention to perform FGM/C on their daughters [22], whilst a 2015 study from Norway among young Somalis living in Oslo, found that both boys and girls perceived it as a harmful practice that should be discontinued [23]. Further, a qualitative Swedish study from 2008, conducted among Eritrean women who had been subjected to FGM/C prior to migrating to Europe, found that the women were against passing on the practice to their daughters as they did not want to pass on their suffering to their daughters [24]. This study also found that the women had both positive and negative experiences with Swedish healthcare persons in relation to childbirth. Whilst some women found that both doctors and midwives had good knowledge of FGM and how to handle it in relation to childbirth, others expressed anxiety and fear due to the lack of ability among healthcare personnel to properly deal with FGM/C in relation to childbirth [24]. Our study also found that study participants felt that Danish healthcare persons did not know of how to properly deal with FGM, however, this was not in relation to childbirth but rather how to properly address and discuss sexual health issues in a wider context.

## 5 Conclusions

This qualitative study found that FGM/C is negatively perceived and not practiced among Somali and Kurdish immigrants and descendants in Denmark, and that Somali immigrants feel stigmatised when it comes to the practice as they are falsely associated with it. These findings are in line with other studies from Europe. Further, our study found that the Danish healthcare system should adopt a more culturally sensitive approach when addressing sensitive issues such as sexual health among immigrants and descendants in Denmark. Denmark and other European countries should work towards destigmatising immigrant communities when it comes to FGM/C.

## Funding

The data was financed by and prepared for the use of the European Institute for Gender Equality (EIGE). Neither the European Institute for Gender Equality nor any person acting on its behalf may be held responsible for the use pertaining to the information contained in this publication. The contents of this publication do not necessarily reflect the position or opinion of the European Institute for Gender Equality.

## CRediT authorship contribution statement

**Ditte Søndergaard Linde:** Writing – review & editing, Writing – original draft, Visualization, Validation, Supervision, Software, Resources, Project administration, Methodology, Investigation, Funding acquisition, Formal analysis, Data curation, Conceptualization. **Hawa-Idil Harakow:** Writing – review & editing, Writing – original draft, Validation, Methodology, Formal analysis, Data curation. **Negin Jaafar:** Writing – review & editing, Writing – original draft, Methodology, Investigation, Data curation.

## Declaration of Competing Interest

There are no conflicts of interest among the authors. The first author was contacted by EIGE and invited to conduct the study as national researcher for Denmark based on her expertise within sexual and reproductive health and accepted to do the study out of research interests.
